# Transcriptomic Changes in the Myocardium and Coronary Artery of Donation after Circulatory Death Hearts following Ex Vivo Machine Perfusion

**DOI:** 10.3390/ijms25021261

**Published:** 2024-01-19

**Authors:** Lars Saemann, Kristin Wächter, Adrian-Iustin Georgevici, Sabine Pohl, Fabio Hoorn, Gábor Veres, Sevil Korkmaz-Icöz, Matthias Karck, Andreas Simm, Gábor Szabó

**Affiliations:** 1 Department of Cardiac Surgery, University Hospital Halle (Saale), University of Halle, 06120 Halle (Saale), Germany; 2 Department of Cardiac Surgery, University Hospital Heidelberg, 69120 Heidelberg, Germany; 3 Department of Anaesthesiology, St. Josef Hospital, Ruhr-University Bochum, 44791 Bochum, Germany

**Keywords:** transcriptomics, microarrays, machine learning, heart transplantation, donation after circulatory death, left anterior descending, senescence induction

## Abstract

Donation after circulatory death (DCD) hearts are predominantly maintained by normothermic blood perfusion (NBP). Nevertheless, it was shown that hypothermic crystalloid perfusion (HCP) is superior to blood perfusion to recondition left ventricular (LV) contractility. However, transcriptomic changes in the myocardium and coronary artery in DCD hearts after HCP and NBP have not been investigated yet. In a pig model, DCD hearts were harvested and maintained for 4 h by NBP (DCD-BP group, *N* = 8) or HCP with oxygenated histidine–tryptophane–ketoglutarate (HTK) solution (DCD-HTK, *N* = 8) followed by reperfusion with fresh blood for 2 h. In the DCD group (*N* = 8), hearts underwent reperfusion immediately after procurement. In the control group (*N* = 7), no circulatory death was induced. We performed transcriptomics from LV myocardial and left anterior descending (LAD) samples using microarrays (25,470 genes). We applied the Boruta algorithm for variable selection to identify relevant genes. In the DCD-BP group, compared to DCD, six genes were regulated in the myocardium and 1915 genes were regulated in the LAD. In the DCD-HTK group, 259 genes were downregulated in the myocardium and 27 in the LAD; and 52 genes were upregulated in the myocardium and 765 in the LAD, compared to the DCD group. We identified seven genes of relevance for group identification: *ITPRIP*, *G3BP1*, *ARRDC3*, *XPO6*, *NOP2*, *SPTSSA*, and *IL-6*. NBP resulted in the upregulation of genes involved in mitochondrial calcium accumulation and ROS production, the reduction in microvascular endothelial sprouting, and inflammation. HCP resulted in the downregulation of genes involved in *NF-κB-*, *STAT3-*, and *SASP*-activation and inflammation.

## 1. Introduction

The transplantation of hearts donated after circulatory death is increasingly performed worldwide to treat end-stage heart failure and increase the donor pool [1]. In the clinical setting, donation after circulatory death (DCD) hearts are predominantly maintained by ex situ normothermic blood perfusion (NBP) in a beating state [2]. NBP facilitates the option to determine the metabolic state and visually inspect the contraction of the heart, which had to withstand a warm ischemic period in the donor body before harvesting [3]. However, we have shown in an experimental model of porcine DCD that oxygenated hypothermic crystalloid perfusion (HCP) with histidine–tryptophane–ketoglutarate (HTK) solution can recondition left ventricular contractility and relaxation [4]. HCP of cardiac allografts does not facilitate the option to inspect contractility visually nor to monitor lactate concentrations in the perfusate. Accordingly, we developed a method to predict left-ventricular contractility after HCP based on microvascular flow shifts during HCP to pave the way for translation into the clinical setting. 

Transcriptomics allows for the studying of all RNA molecules within a tissue or cell of interest to determine a gene expression signature or to potentially unravel the molecular etiology for diseases, phenotypes, or functional differences in tissues. Although heart transplantation (HTX) from DCD is increasingly performed and shows promising short-term results, a transcriptomic signature does not exist yet. Furthermore, potential transcriptomic changes that might explain functional differences in the left ventricular performance of DCD hearts after NBP or HCP have not been investigated yet [2]. However, it is known that NBP and HCP lead to differing left ventricular systolic and diastolic functions of DCD hearts [4,5]. Thus, we performed microarray analyses of the left ventricular myocardium and left anterior descending (LAD) coronary artery of hearts after circulatory death induction and of DCD hearts that were preserved by NBP and HCP.

## 2. Results

### 2.1. Effect of Circulatory Death Induction

**Figure 1:**
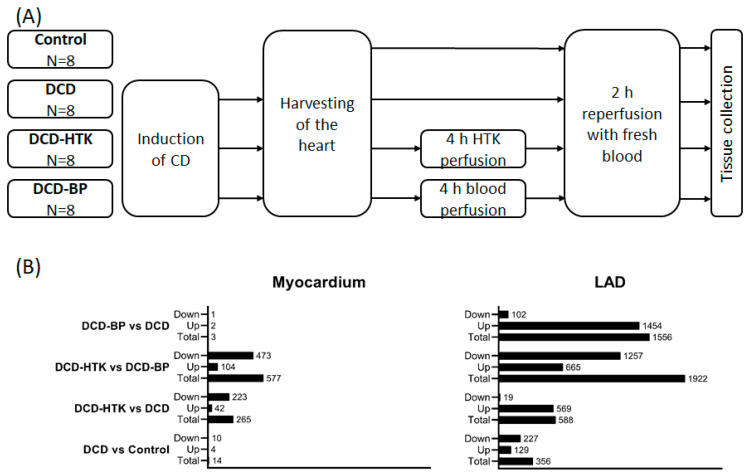
Overview. (**A**) Groups. (**B**) Gene expression. BP: blood perfusion. DCD: donation after circulatory death. HTK: histidine–tryptophane–ketoglutarate.

**Figure 2:**
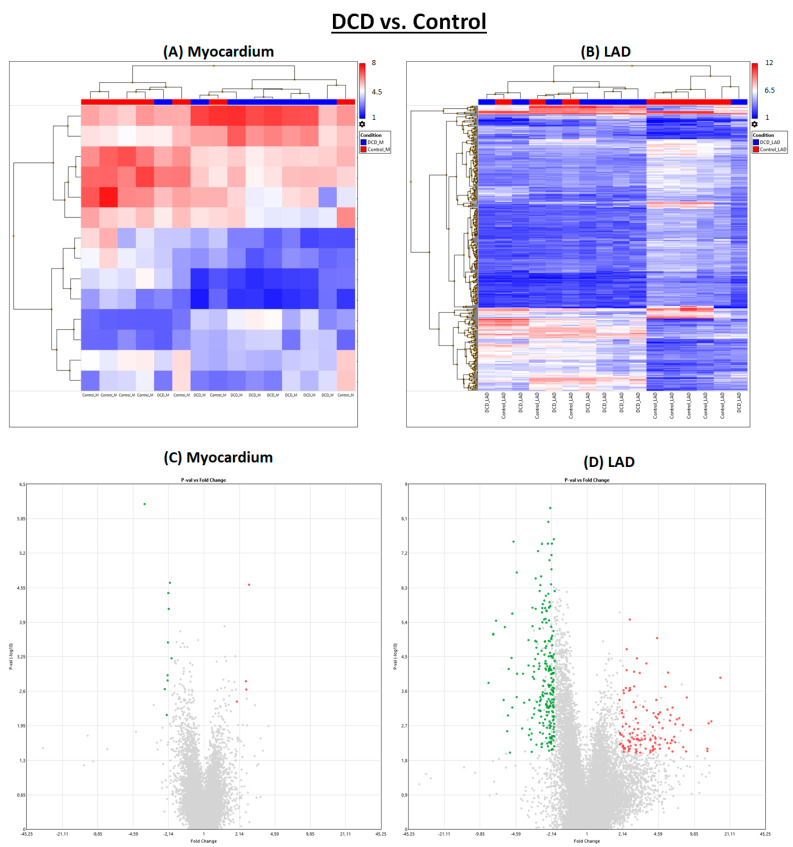
DCD vs. control hearts. (**A**,**B**) Heat maps. (**C**,**D**) Volcano plots. Green: significantly downregulated genes. Red: significantly upregulated genes. Grey: Not significantly regulated and/or not regulated > 2 or <−2 fold change. DCD: donation after circulatory death. LAD: left anterior descending.

Induction of circulatory death resulted in noticeably more significantly regulated genes in LAD than in myocardial tissue (Figure 1 and Figure 2). 

### 2.2. Effect of Normothermic Blood Perfusion

**Figure 3:**
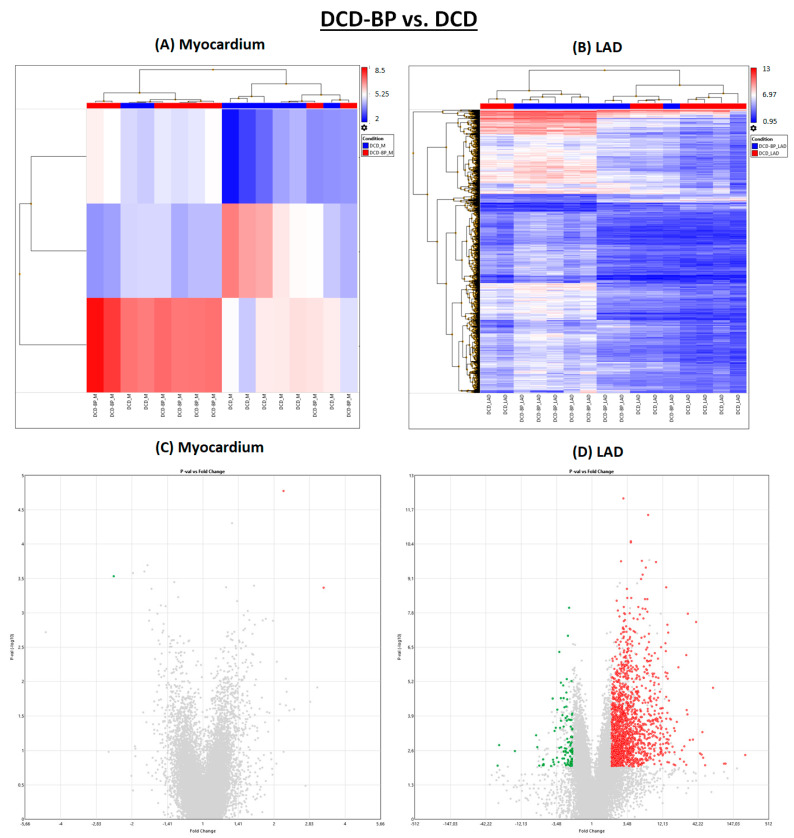
DCD-BP vs. DCD. (**A**,**B**) Heat maps. (**C**,**D**) Volcano plots. Green: significantly downregulated genes. Red: significantly upregulated genes. Grey: Not significantly regulated and/or not regulated > 2 or <−2 fold change. BP: blood perfusion. DCD: donation after circulatory death. LAD: left anterior descending.

After NBP, individual subjects of the DCD group and the DCD-BP group aligned well next to each other (Figure 3). Only three genes were regulated in the myocardium in the DCD-BP group compared to the DCD group. However, 1556 genes in total were regulated in the LAD (Figure 1).

### 2.3. Effect of Hypothermic Crystalloid Perfusion

**Figure 4:**
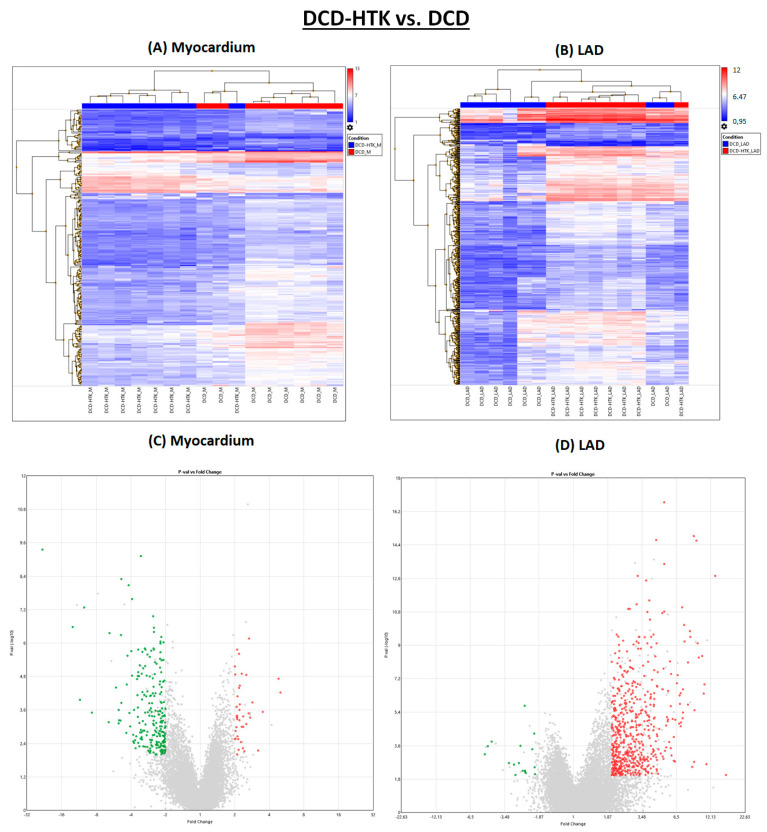
DCD-HTK vs. DCD. (**A**,**B**) Heat maps. (**C**,**D**) Volcano plots. Green: significantly downregulated genes. Red: significantly upregulated genes. Grey: Not significantly regulated and/or not regulated > 2 or <−2 fold change. DCD: donation after circulatory death. LAD: left anterior descending. HTK: histidine–tryptophane–ketoglutarate.

Different from NBP, after perfusion with HTK, in the myocardium, 223 genes were downregulated and 19 genes were downregulated in the LAD (Figure 1 and Figure 4), compared to the DCD group. In contrast, 42 genes were upregulated in the myocardium and 569 in the LAD of the DCD-HTK group compared to the DCD group. 

### 2.4. Hypothermic Crystalloid Perfusion vs. Normothermic Blood Perfusion

**Figure 5:**
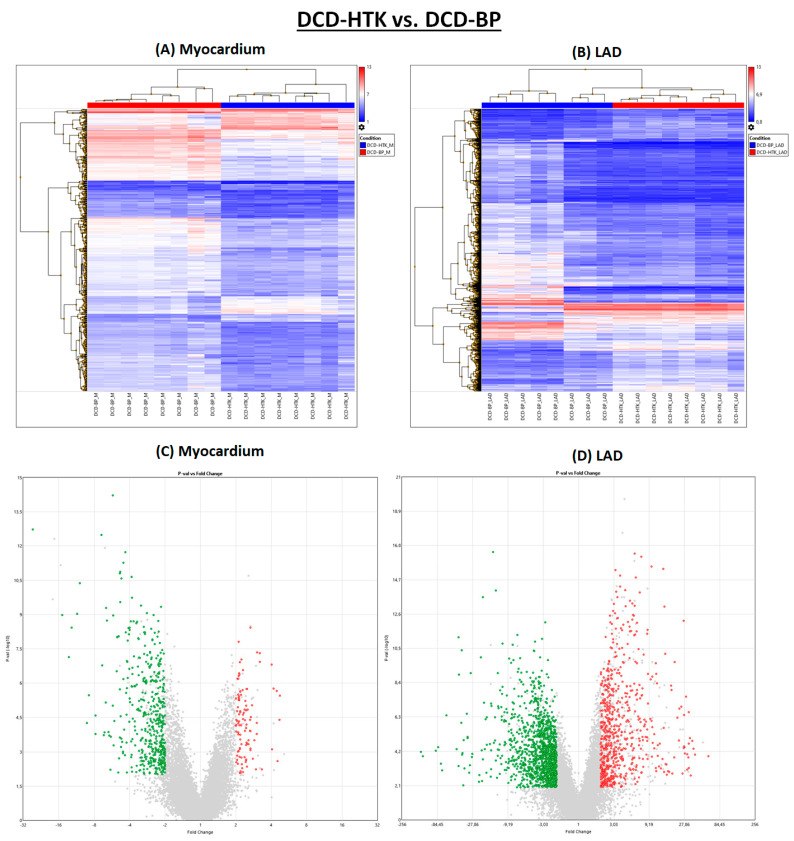
DCD-HTK vs. DCD-BP. (**A**,**B**) Heat maps. (**C**,**D**) Volcano plots. Green: significantly downregulated genes. Red: significantly upregulated genes. Grey: Not significantly regulated and/or not regulated > 2 or <−2 fold change. BP: blood perfusion. DCD: donation after circulatory death. LAD: left anterior descending. HTK: histidine–tryptophane–ketoglutarate.

In the DCD-HTK group compared to the DCD-BP group, 473 genes were downregulated and 104 genes were upregulated in the myocardium, and 1257 genes were downregulated and 665 genes were upregulated in the LAD (Figure 1). In the heat maps, the groups were clearly separated from each other (Figure 5).

### 2.5. Network Analysis

**Figure 6:**
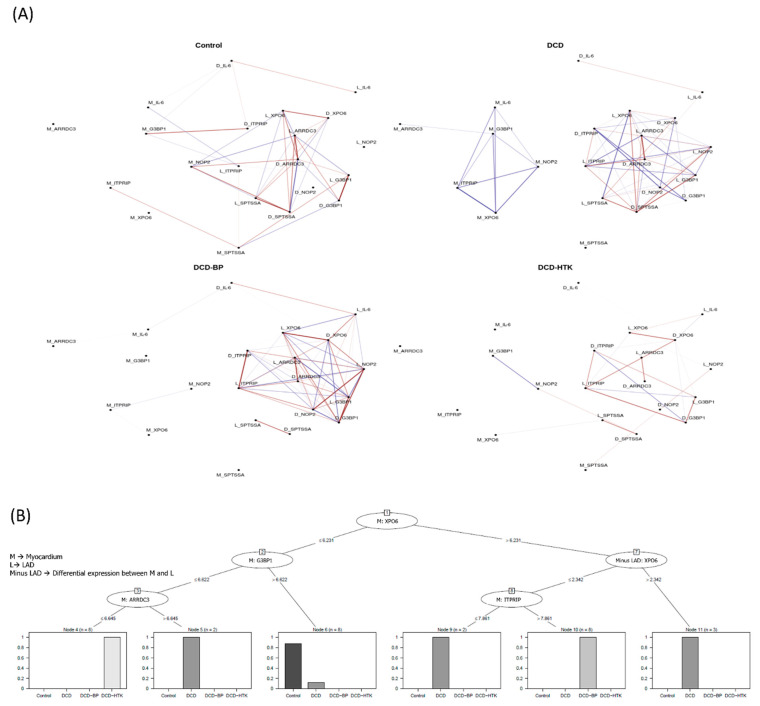
Machine learning analysis. (**A**) Network analysis. (**B**) Decision tree analysis. BP: blood perfusion. DCD: donation after circulatory death. LAD: left anterior descending. HTK: histidine–tryptophane–ketoglutarate. M, L, and D combined with gene symbols reflect the myocardial expression, LAD expression, or expression difference between both tissues.

The network analysis (Figure 6) shows a significant correlation between relevant genes. Seven genes were identified: inositol 1,4,5-trisphosphate receptor-interacting protein *(ITPRIP)*, GTPase activating protein (SH3 domain) binding protein 1 *(G3BP1)*, arrestin domain containing 3 *(ARRDC3)*, exportin 6 *(XPO6)*, NOP2 nucleolar protein *(NOP2)*, serine palmitoyltransferase, small subunit A *(SPTSSA)*, and interleukin-6 *(IL-6)*.

In the DCD group, two clusters are visible. One cluster includes only positive correlation of the myocardial expression of *ITPRIP*, *G3BP1*, *ARRDC3*, *XPO6*, *NOP2*, and *IL-6*. The second cluster comprises positive and negative correlations, consisting of the expression of *XPO6*, *ITPRIP*, *SPTSSA*, *OP2*, *G3BP1*, and *ARRDC3* in the LAD and the differential expression between the myocardium and LAD.

After NBP and HCP, the cluster consisting of solely positively correlated genes no longer exists. However, in the DCD-BP and DCD-HTK groups, the expression of *SPTSSA* in the LAD and the differential expression between the myocardium and LAD also correlate with each other, but both do not correlate anymore with the main network. The correlation of *G3BP1* in the LAD and the differential expression between the myocardium and LAD are existent in the control group hearts but not in the DCD group and were reconstituted in both DCD-BP and DCD-HTK.

Neither in the DCD group nor in the DCD-BP group is the myocardial expression of *SPTSSA* connected with the main network. Only in the DCD-HTK group is the myocardial expression of *SPTSSA* connected to the main network, as well as in the control group hearts.

However, not all correlations of the network in the control group are visible in the DCD-HTK group. Thus, the correlation of *IL-6* in the LAD and the differential expression between the myocardium and LAD exist in all but the DCD-HTK group, while the correlation of *IL-6* in the myocardium and *ITPRIP* in the LAD, as well as of *G3BP1* in the myocardium and the differential expression of *ITPRIP* in the myocardium and the LAD, exists only in the control group.

### 2.6. Decision Trees

We identified the expression of five distinct genes to be relevant in a hierarchical manner for differentiation between individual groups (Figure 6):

(1) The myocardial regulation of *XPO6* and *G3BP1* is characteristic in native control hearts.
(2) The regulation of *XPO6* followed by *G3BP1* and *ARRDC3*, all in the myocardium, is characteristic in DCD hearts preserved by HTK perfusion.
(3) The regulation of *XPO6* followed by the differential expression of *XPO6* in the myocardium compared to LAD and the regulation of ITPRIP in the myocardium are characteristic of DCD hearts preserved by blood perfusion.

## 3. Discussion

It is known from the literature that HCP with HTK solution is superior to NBP, not only to maintain but also to recondition left ventricular contractility of DCD hearts in a porcine model [4]. However, comprehensive mechanistic insights regarding gene expression signatures were missing until now. 

Induction of circulatory death and the type of machine perfusion, NBP or HCP, lead to distinct gene expression signatures that differ widely. In total, more genes are affected in the LAD than in the myocardium. However, mainly the regulation of myocardial expression of distinct genes allows for clear differentiation between the individual groups. 

**Figure 7:**
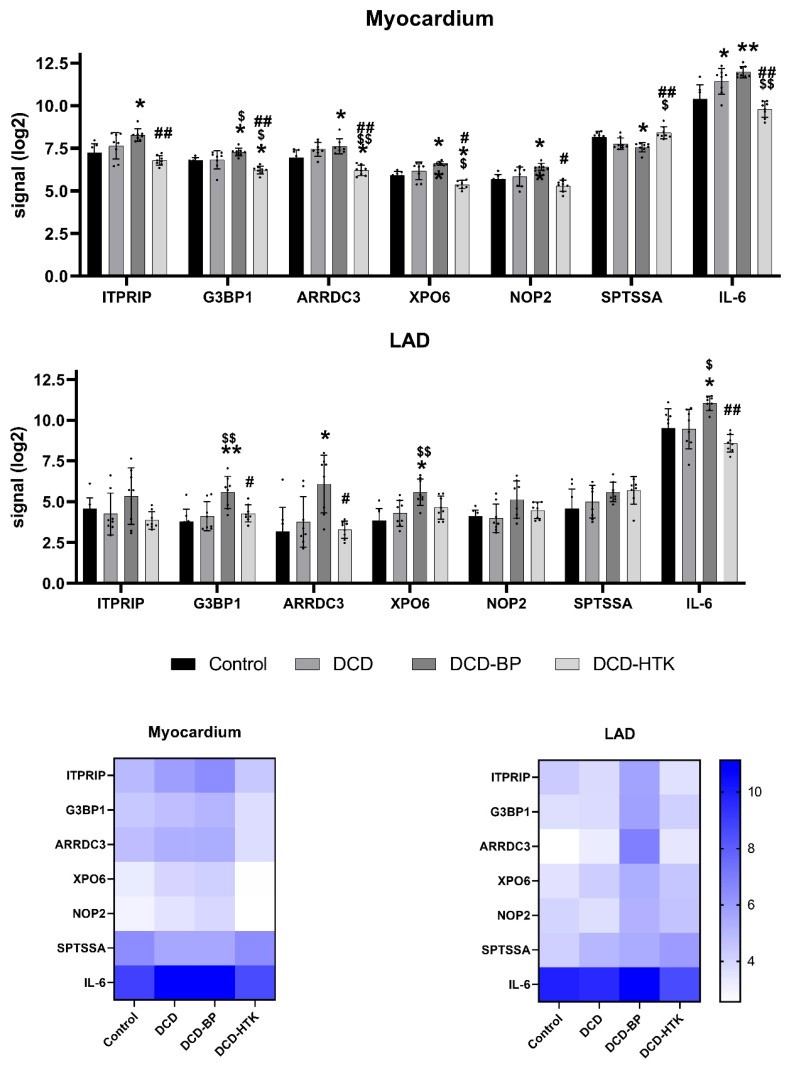
Expression of key genes. *, ** *p* < 0.05 or <0.001 vs. control. ^$^, ^$$^ *p* < 0.05 or <0.001 vs. DCD. ^#, ##^ *p* < 0.05 or <0.001 vs. DCD-BP.

ITPRIP enhances the sensitivity of the inositol 1,4,5-trisphosphate receptor (ITPR) to intracellular calcium signaling and endoplasmatic reticulum calcium release through ITPR2 channels and leads to mitochondrial calcium accumulation. Furthermore, it induces reactive oxygen species (ROS) accumulation. Wiel et al. have also shown in their experiments that this ROS accumulation is associated with the induction of senescence, concluding that ITRIP is also involved in the induction of senescence [6]. *ITRIP* was significantly upregulated in the DCD-BP group (Figure 7), which might explain the decreased contractility that we observed in the previously published results [4]. Interestingly, circulatory death induction in the LAD did not lead to an upregulation of *ITRIP*.

*ARRDC3* was upregulated in the DCD-BP group, especially in the LAD, and reduced microvascular endothelial sprouting during hypoxia, as shown by Nauta et al. [7]. Considering this, the difference in coronary microvascular circulation after NBP and HCP should be compared. SPTSSA stimulates the serine palmitoyltransferase, and Lee et al. demonstrated that a cardiomyocyte-specific deficiency of the serine salmitoyltransferase subunit 2 leads to cardiac dysfunction [8]. Thus, it can be assumed that the downregulation of *SPTSSA*, visible in the myocardium of DCD and DCD-BP hearts, might also provoke cardiac dysfunction. *SPTSSA* was downregulated by circulatory death induction, and the expression remains unchanged during NBP. G3BP1, which was downregulated in both the myocardium and LAD in the DCD-HTK group and significantly upregulated in the DCD-BP group, promotes the association of the cyclic GMP-AMP synthase (cGAS) with cytosolic chromatin fragments. Second, G3BP1 activates the NF-κB and STAT3 pathways through cGAS, releasing specific profile of molecules known as the senescent-associated secretory phenotype (SASP) [9]. The SASP is usually released from old, senescent cells and consists of a wide range of proinflammatory cytokines and matrix-degrading factors reducing the viability of non-senescent neighboring cells.

Consequently, using HCP could be even more beneficial for maintaining hearts donated by old donors. *G3BP1* was not increased in the myocardium of DCD hearts without subsequent NBP. Thus, we conclude that NBP, but not circulatory death induction, triggers the upregulation of this *G3BP1*. *IL-6*, a critical proinflammatory cytokine, was downregulated in the DCD-HTK group in the myocardium and LAD. This suggests potential anti-inflammatory effects of HCP with HTK compared to NBP. Interestingly, *IL-6* was already upregulated by circulatory death induction in the myocardium but not in the LAD. It is commonly upregulated after ischemia/reperfusion, is involved in allograft injury, plays a key role in regulating the inflammatory and alloimmune responses, and finally, leads to poor long-term survival after HTX [10].

DCD-HTK and control hearts have in common that the same regulation of *XPO6* is characteristic. Exportins are proteins that transport macromolecules from the nucleus into the cytoplasm. Nevertheless, currently, nothing is known about the role of XPO6. Thus, an interpretation of the expression of *XPO6* based on the literature is not possible. For the DCD and DCD-BP groups, the differential expression of *XPO6* in the myocardium compared to LAD was identified to be highly relevant. This shows that the expression of *XPO6* in the myocardium and the LAD of the groups DCD and DCD-BP does not change in parallel in all groups.

NOP2 is involved in the positive regulation of cell population proliferation. Nevertheless, nothing is known about its particular function in cardiac tissue. The role of XPO6 and NOP2 in the myocardium and coronary artery should be investigated.

HCP and NBP differ profoundly in their composition as well as by temperature. The HTK solutions contain different buffer substances, such as histidine, α-ketoglutarate, tryptophan, and the oncotic agent mannitol [5]. In addition to the low temperature, the cardioplegic properties of the HTK solution result in transportation in a non-beating mode. Thus, the first hours of reperfusion are also in non-beating mode during HCP, compared to beating mode in NBP. The cardioplegic properties are based on the low sodium concentrations of the HTK solution.

Furthermore, calcium is depleted in the HTK solution, while NBP is normocalcemic. NBP was discussed to be more physiologic than HCP. However, during NBP, the heart is continuously perfused with blood from the ischemic DCD donor, which contains proinflammatory cytokines. Additionally, beating empty over several hours induces apoptosis in the cardiomyocytes and promotes edema formation. Finally, the combination of hypothermic conditions, induced cardiac arrest, oxygen, and buffer substance supply seem to result in functional improvements [4] and molecular mechanistic changes.

The gene expression signatures accord to the already known histological findings, such as decreased oxidative and nitrosative stress and less edema formation in the HTK group [4].

For the effective treatment of end-stage heart failure, both short-term and long-term outcomes are important. In this transplantation-equivalent model, short-term results that refer to a time point immediately after transplantation can be investigated. Nevertheless, the molecular insights also allow for suggestions regarding the long-term outcome. The long-term outcome is especially dependent on the development of cardiac allograft vasculopathy (CAV). Ischemia/reperfusion-related vascular endothelial injury and vascular inflammation of the coronary arteries are significant triggers for the development of CAV [11]. Among the six identified genes with potential key functions already discussed above, three are associated with ischemia/reperfusion-related vascular injury and inflammation: ARRDC3, G3BP1, and IL6. G3BP1 activates two pathways, NF-κB and STAT3, that lead to the release of a whole group of proinflammatory cytokines and other molecules known as the SASP. The correlation between SASP and long-term graft failure has not been investigated yet. However, a causative connection is likely, based on the current literature [12].

## 4. Materials and Methods

### 4.1. Animals and Anesthesia

The investigations were reviewed and approved (35-9185.81/G-150/19) by the appropriate institutional Ethical Committee for Animal Experimentation. The animals received human care. We sedated healthy pigs (35–40 kg bodyweight) with an intramuscular injection of ketamine (22.5 mg/kg; Bremer Pharma, Warburg, Germany) and midazolam (0.375 mg/kg; Hameln pharma plus, Hameln, Germany) as described in detail previously [13]. For the maintenance of anesthesia, we delivered pentobarbital sodium intravenously through the ear vein (15 mg/kg/h; Boehringer Ingelheim Vetmedicia, Ingelheim, Germany). Dipidolor (1.125 mg/kg/h; Piramal Critical Care, Voorschoten, The Netherlands) was used for analgesia. We monitored the blood pressure and performed blood sampling through arterial and venous vascular access in the femoral artery and vein. Based on blood gas analysis, we adjusted the partial pressure of oxygen (paO_2_) to 200 mmHg and the partial pressure of carbon dioxide (paCO_2_) to 35–45 mmHg. 

### 4.2. Study Groups and Donation after Circulatory Death Model

We opened the chest by median sternotomy and exposed the heart. Then, we injected heparin (LEO Pharma, Neuisenburg, Germany) intravenously to achieve systemic anticoagulation. The study included four groups: In the control group (control group; *N* = 8), native control hearts were cardioplegically arrested with 2 L of cold (4 °C) Custodiol^®^ organ preservation solution (Köhler Chemie GmbH, Bensheim, Germany) and harvested (Figure 1). In the DCD group (DCD; *N* = 8), circulatory death was induced by the termination of mechanical ventilation [4]. After a total warm ischemic time of 30 min, we also flushed the DCD hearts with 2 L of cold (4 °C) Custodiol^®^ organ preservation solution. In two other groups, after harvesting, we maintained the DCD hearts for 4 h by HCP with HTK (DCD-HTK group, *N* = 8) solution at 4 °C or by NBP (DCD-BP group; *N* = 8) at 37 °C [4]. During NBP, we added heparin (5000 iU), sodium chloride, magnesium chloride, glucose, sodium–prednisolone, sodium hydrogen carbonate, and mannitol to the perfusion system. The partial pressure of oxygen was maintained at 180–200 mmHg, and the partial pressure of carbon dioxide was adjusted to 35–45 mmHg. We adjusted the pH to 7.35–7.45. We applied a pressure-controlled perfusion regimen with a perfusion pressure of 60 mmHg. We measured arterial and venous blood gas every 30 min with a point-of-care analyzer (RAPID Point 500, Siemens, Erlangen, Germany).

### 4.3. Reperfusion with Blood

In all groups, hearts were reperfused for 2 h with fresh oxygenated blood to mimic transplantation. During reperfusion, we adjusted blood gas parameters according to physiological ranges as described for NBP.

### 4.4. Tissue Collection

At the end of reperfusion, we flushed the hearts with an ice-cold Ringer solution. Myocardial tissue samples were immediately snap frozen in liquid nitrogen. The LAD, including surrounding myocardial tissue, was quickly excised and placed in an ice-cold carbogenized Krebs–Henseleit buffer solution. Under the microscopic vision, the LAD was carefully but quickly dissected and snap frozen in liquid nitrogen. All tissue samples were stored at −80 °C until further investigation.

### 4.5. RNA Preparation

Total RNA was isolated from the left ventricular myocardium and LAD of every pig (*N* = 8 per group; *N* = 32 in total). RNA was isolated by TRIzol (Thermo Fisher Scientific, Waltham, MA, USA) extraction. Therefore, the samples were homogenized using a Tissue Lyser II (Qiagen, Hilden, Germany). Then, chloroform was added to induce phase separation. After centrifugation, the upper phase was agitated by incubation with isopropanol. Then, we pelletized the RNA by centrifugation at 4 °C and washed the pallet with NaAc. Then, the pellet was dissolved overnight at −20 °C in DEPC-H_2_O, followed by two washing steps with 80% ethanol. Then, the RNA was stored in DEPC-H_2_O at −80 °C.

### 4.6. Microarrays

First, we assessed the RNA integrity using a bioanalyzer (2100 Bioanalyzer, Agilent, Santa Clara, CA, USA). We determined the RNA concentration using Nanodrop One (Thermofisher, Waltham, MA, USA). Biotin-labeled ss-cDNA was synthesized from total RNA with a GeneChip™ WT Pico Reagent Kit (Thermo Fisher Scientific, Waltham, MA, USA), fragmented, and subsequently hybridized using porcine arrays (Thermo Fisher Scientific, Waltham, MA, USA). Afterward, the chips were washed and scanned by the Affymetrix GeneChip Scanner 7G (Thermofisher, Waltham, MA, USA). One control group sample was later identified as an outlier and therefore excluded from the analysis.

### 4.7. Statistical Analysis and Machine Learning Algorithm

Heat maps and volcano plots were built using the Transcriptome Analysis Console (TAC 4.0; Applied Biosystems; Thermo Fisher Scientific, Waltham, MA, USA). Differentially expressed genes were displayed through fold change (upregulated > 2.0; downregulated < −2.0), together with a *p*-value < 0.01 using eBayes statistics. 

Next to the measured gene expression (GE) from myocardium and LAD, we also computed per animal the difference between the former and latter: GE_MminusLAD_ = GE_myocardium_ − GE_LAD_. In the first step, we pre-filtered the GE with significant *p*-values (<0.05) across the DCD, DCD-HTK, and DCD-BP groups using Kruskal–Wallis, a non-parametric test not assuming Gaussian distribution. All three types of GE were tested: myocardial, LAD, and MminusLAD. The gene was included in the further analysis if the GE was significantly regulated in at least one of those three. In the second step, we performed variable selection applying the Boruta performant machine learning method for variable selection to identify the GE that is informative for the classification of the study groups. The Boruta algorithm uses ensembles of decision trees to estimate non-parametrically multivariate and non-linear associations. As such, a variable is confirmed as informative by the Boruta algorithm if the variable importance distribution is statistically significant at the Bonferroni-corrected *p*-value < 0.01. A total of 1000 Boruta iterations were computationally allowed. normHits represents the percent of iterations where the variable importance of data is higher than its permutation [14]. We explored how multiple variables separate across study groups using the C.50 tree algorithm, a method for interpretable machine learning [15]. Finally, we explored the global multivariate patterns by comparing differences across the architectures of the GE networks. The network’s nodes are variables confirmed in both statistical and machine learning steps. The edges between nodes are the absolute values of the Pearson correlation coefficients if the FDR-adjusted *p*-values are significant. The graph was computed using the stress majorization layout [16].

## 5. Conclusions

Induction of circulatory death and the type of ex vivo machine perfusion significantly impact gene regulation in both the left ventricular myocardium and LAD in a porcine model, leading to individual gene expression signatures. However, the gene expression change by circulatory death induction or machine perfusion in the myocardium differs from that in the LAD. NBP resulted in the upregulation of genes involved in mitochondrial calcium accumulation and ROS production, inflammation and reduced microvascular endothelial sprouting, and the downregulation of genes essential for cardiac function. HCP with HTK resulted in downregulating genes involved in NF-κB, STAT3, and SASP activation, inflammation, allograft injury, and primary graft dysfunction. Thus, the identified gene expression signatures explain the already known superior contractility of hearts maintained by HCP with HTK compared to NBP [4]. Considering both known functional differences and novel mechanistic insights, we conclude from our results that HCP with HTK is superior to NBP in maintaining and protecting DCD hearts during transportation.

## Data Availability

Data will be provided on reasonable request.

## Abbreviations

ARRDC3	Arrestin domain containing 3
CAV	Cardiac allograft vasculopathy
DCD	Donation after circulatory death
G3BP1	GTPase activating protein (SH3 domain) binding protein 1
GE	Gene expression
HCP	Hypothermic crystalloid perfusion
HTK	Histidine–tryptophane–ketoglutarate
HTX	Heart transplantation
IL-6	Interleukin-6
ITPR	Inositol 1,4,5-trisphosphate receptor
ITPRIP	Inositol 1,4,5-trisphosphate receptor interacting protein
LAD	Left anterior descending
NBP	Normothermic blood perfusion
NOP2	NOP2 nucleolar protein
ROS	Reactive oxygen species
SASP	Senescent-associated secretory phenotype
SPTSSA	Serine palmitoyltransferase, small subunit A
XPO 6	Exportin 6
